# COMBINED INSIDE-OUT AND ALL-INSIDE TECHNIQUE IN BUCKET-HANDLE MENISCUS TEARS

**DOI:** 10.1590/1413-785220162404156575

**Published:** 2016

**Authors:** Serdar Yılmaz, Deniz Cankaya, Ahmet Fırat, Alper Devecı, Bulent Ozkurt, Murat Bozkurt

**Affiliations:** 1. Ankara Numune Training and Research Hospital, Department of Orthopaedics and Traumatology, Ankara, Turkey.; 2. Ankara Ataturk Training and Research Hospital, Department of Orthopaedics and Traumatology, Ankara, Turkey.

**Keywords:** Menisci, tibial/injuries, Knee, Arthroscopy

## Abstract

**Objective:**

To determine the outcomes after combined inside-out and all-inside repair technique of bucket-handle meniscus tears*.*

**Methods:**

A retrospective review was made of patients with bucket-handle meniscus tears repaired with combined techniques, using the all-inside technique in posterior meniscal tears and the inside-out technique in the middle part of the meniscal tears. Meniscal healing was assessed clinically using Barrett's criteria and MRI*.*

**Results:**

The study comprised 52 patients with a mean age of 28.4 years old (range, 19-52 years old). The mean follow-up period was 31.3 months (range, 24-59 months). Two patients had ACL re-rupture, and complete meniscal healing was achieved in all but one patient. Although improved from preoperative status, Tegner and Lysholm scores were lower in the ACL reconstructed patients than in the intact ACL patients*.*

**Conclusion:**

Combined inside-out and all-inside meniscal repair technique is a successful and cost-effective treatment method in bucket-handle meniscus tears. *Level of Evidence IV, Therapeutic Study.*

## INTRODUCTION

The meniscus is a fibrocartilaginous tissue, which is essential in the knee joint. Due to the critical role in knee biomechanics, meniscal injuries may lead to long-term degenerative joint changes. As arthroscopic techniques and instrumentation have advanced, so treatment has been focused on meniscal repair rather than meniscectomy.^1^ ^,^ ^2^

The treatment of meniscal injuries depends on various factors such as the localization, type of the tear and the age of the patient.^2^ ^-^ ^5^ Meniscal tears must be repaired whenever possible because of the critical role in knee biomechanics. There are several well-known techniques for repairing the meniscus: inside-out, outside-in and all-inside techniques.^1^ ^,^ ^2^ ^,^ ^6^ ^,^ ^7^ At present, it can be concluded that no single meniscal repair technique or device is superior in all situations, so there is no consensus on the ideal suture technique. The inside-out technique is simple and has been used successfully for a long time.^1^ Due to the neurovascular complication risk in the posterior part of meniscal tears, the all-inside technique has been developed.^5^ The all-inside technique has gained in popularity with the

advantages of easy application, there being no need to make an accessory incision, low complication rate and decreased surgery time, but there are also some disadvantages such as higher cost, chondral injury, pain around the implant, synovitis and parameniscal cyst.^1^ ^,^ ^5^ ^,^ ^8^ ^-^ ^11^

A bucket handle meniscal tear (BHMT) has been described as a vertical or oblique tear extending longitudinally and the inner portion is prolapsed into the intercondylar notch.^12^ The treatment of BHMT is important because the torn part is so large that excision may lead to degenerative arthritis. However, the reduction of the meniscal tear is sometimes difficult and can lead to serious problems such as locked knee.^12^ ^,^ ^13^ Therefore, it has been not defined as the best meniscal repair technique in BHMT.

In the current study, these two techniques were combined as a 'combined meniscal repair' (CMR) using the all-inside technique in posterior meniscal tears and the inside-out technique in the middle part of the meniscal tears. The aim of this study was to verify whether the effectivity of this technique and the complication rates in patients who underwent surgery for BHMT. Also we made cost analysis about this combined technique. In addition, a comparison was made of the outcomes of patients with isolated meniscal repairs and those with associated anterior cruciate ligament (ACL) reconstruction.

## MATERIALS AND METHODS

A retrospective evaluation was made of arthroscopically repaired BHMT patients between 2008 and 2012. Approval for the study was granted by the Institutional Review Board and informed consent was obtained from all the participants. Patients were excluded who had any previous meniscal repair surgery or meniscectomy, chronic tears (>12 weeks), horizontal, transverse or complex tears of the meniscus or evidence of arthritis. MRI was applied to all patients before surgery to aid the surgical decision. Meniscal tears in the red-red or red-white zones without obvious degeneration were indicated for meniscal repair. Anterior cruciate ligament (ACL) deficient knees were reconstructed using hamstring autograft or allograft at the time of the meniscal repair with the endobutton technique. According to these criteria, 52 patients (36 male, 16 female) were included in the study. The mean age was 28.4 years (range 19-52 years). Clinical evaluation and the file review was made by an independent orthopaedic surgeon (YS). Patients with a minimum follow-up of 24 months (mean, 31.3 months; range, 24-59 months) were included in this study. Information was obtained from the patient file of the age of the patient, follow-up period, time interval between the injury and surgery, the mechanism of injury, any associated ligamentous or chondral injury, additional surgery, the location of the tear, and the preoperative activity level. The mean interval from the recalled injury to surgery was 14.2 days (range, 3 days to 12 weeks). Six patients involved in professional sports had ACL reconstruction with an allograft and the others had reconstruction with autograft. The demographic data of the patients are shown in Table 1.

**Table 1:** Demographic data of the patients.

Mean age	28.4 (range, 19-52 years)
Gender (male/female)	39/23
Operation time from injury	14.2 days (range, 3 days-3 weeks)
Operation side (right/left)	36/26
Meniscus side Medial Lateral Both meniscus	44 16 2
Location of tear Red-red zone Red-white zone	47 17
The mechanism of injury Soccer injuries Basketball injuries Other sports injuries Jump from height Other	34 12 3 5 8
Associated ACL reconstruction	40

### Surgical technique and rehabilitation protocols

Standard arthroscopy was performed to confirm the presence of a BHMT. Once this tear had been identified and considered appropriate for repair, the tear edges were freshened with a meniscal rasp and shaver. The all-inside technique was used with Ultra FasT-Fix meniscal repair system (Smith & Nephew, Andover, MA, USA) with one or two sutures depending on the tear length in the posterior part of the teared meniscus. If ACL reconstruction was to be performed concomitantly, the meniscal suture was applied before the tibial fixation. The inside-out technique was used with non-absorbable No. 2-0 Ti-cron sutures with the inside-out meniscus repair set (Smith & Nephew, Andover, MA, USA) to the middle part of the meniscal tear. If there was any difficulty during passage of the guide or the suture through the tear such as flip over, initially a horizontal or vertical matrix suture was applied to the junction of the posterior and medial part of the meniscus via the suture guide to facilitate the suturation. Then, the tied suture ends were stretched and in this position sutures were applied to the anterior part of the meniscus tear. The ends of the sutures were taken outside the joint capsule from the mini incision and tied over the capsule. If ACL reconstruction was not considered, microfracture application was made through the intercondylar notch to stimulate the healing response of the meniscus. The ACL reconstruction was done with anatomic one bundle reconstruction with the endobutton technique (endobutton CL, Smith & Nephew).

**Figure 1:**
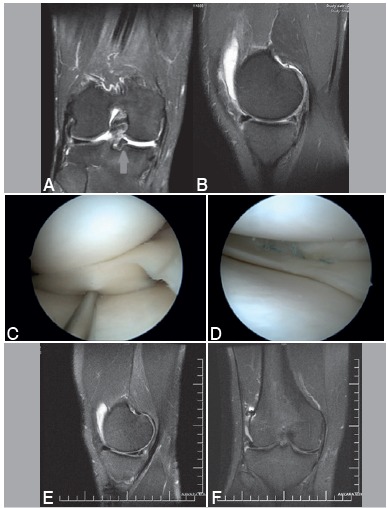
A displaced BHMT associated with ACL tear seen on the coronal (A) and sagittal image (B) of MRI. The displaced meniscal part migrated to the intercondylar notch and remarked with an arrow. The displaced tear was seen on arthroscopy (C). Posterior meniscal tear was sutured with the all-inside technique with 2 sutures. The middle part of the tear was sutured with the inside-out technique (D). The repaired meniscus was almost completely healed at the postoperative 26-month evaluation on sagittal and coronal images of MRI (E, F).

Postoperative rehabilitation was started with the aid of a physiotherapist. In the early period isometric quadriceps exercises were started immediately, as tolerated by the patient. The rehabilitation program was performed together with an ACL rehabilitation program if it had been reconstructed. In this program a hinged knee brace was used to protect the graft from excessive forces and motion was initiated so that 90° knee flexion was accomplished in the postoperative fourth week, after which the brace was discontinued. The patients were started on toe touch crutch walking for 6 weeks. After six weeks the patients were allowed to walk with full weight-bearing. The patients were encouraged to return to their routine activity by 12-20 weeks, but sports activity was restricted for 6 months. An accelerated program of exercise was used in meniscus repairs without ACL surgery, with restriction of motion up to 90º for 4 weeks, no postoperative bracing, restricted rotational and pivoting movements of the knee and toe touch weight-bearing for 6 weeks. (Figures 1 and ^2^)

**Figure 2:**
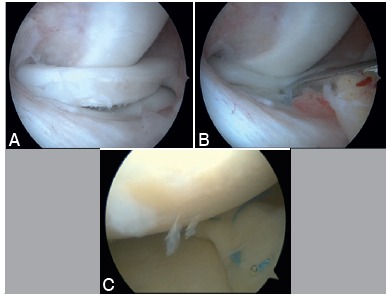
The displaced BHMT seen in the medial meniscus (A) and reduced with a probe (B) on arthroscopy. Combined repair was made via vertical matrix suture applied to the junction of the posterior and medial part of the meniscus to facilitate the repair. Two all-inside sutures were applied and one inside-out suture was applied to the middle part of the tear (C).

An experienced surgeon (YS) performed all the postoperative examinations. The repaired meniscus was considered healed according to both Barrett's criteria and MRI. Using Barrett's criteria,^3^ a repaired meniscus was considered healed if there was no joint-line tenderness or effusion and a negative McMurray test at the final follow-up. The meniscus was considered healed if there was no fluid signal within the meniscus or the signal approached only one articular surface. If the signal hyperintensity extended from one articular surface to the other, the meniscus was considered unhealed.^14^ If one or more of these parameters was present, the result was classified as a failure. Knee stability was evaluated using Lachman and pivot-shift tests. The knee function in daily living activities and in recreational and competitive sports was assessed using the Lysholm and Tegner activity scores. Radiographic evaluation of the healing state of the meniscus and ACL was made with MRI at the final follow-up. The cost of the sutures used for BHMT was also calculated.

### Analyse statistics

The mean value for quantitative variables was expressed as mean and standard deviation. The paired t test and Wilcoxon signed rank test were used for the comparison of the preoperative and postoperative Lysholm and Tegner scores. Comparisons between associated ACL reconstructed and isolated tears were performed using the Fisher Exact test as appropriate. Statistical significance was set at the level of p<0.05.

## RESULTS

The medial meniscus was involved in 36 patients (69.2%) and the lateral meniscus was involved in 14 patients (26.9%). Two patients had both medial and lateral meniscal repair (3.9%). Forty-two meniscal tears (77.8%) were within the red-white zone and 12 tears (22.2%) were within the red-red zone. Thirty-four patients (65.4%) had anatomic one bundle ACL reconstruction concomitantly. Three patients had Grade 1 medial collateral ligament injury and were treated conservatively.

The mean follow-up period was 31.3 months (range, 16 to 59 months). No patient demonstrated joint line tenderness or complained of pain or click on the McMurray test. All the meniscal tears were healed at follow-up MRI. However, 2 patients were admitted after minor trauma during follow-up period. ACL re-rupture was found in the patient who had simple fall 3 months after surgery with healed meniscus and had revision ACL reconstruction with allograft. ACL elongation and unhealed meniscus tear was found in the second patient who played football 7 months after surgery. This patient had also revision ACL reconstruction with allograft and partial meniscectomy. The Lachman test was Grade 1 in 5 patients but they had no complaints of the knee giving way. The pivot-shift test was negative in all but 2 patients with ACL re-rupture. The average number of sutures used for repair was 3.2 (range, 2-5) (1.2 for inside-out and 2 for all-inside). There were no cases of infection. The cost of the FasT-Fix meniscal repair system which was used for the all-inside technique was found to be $335 per patient, whereas the Ti-cron sutures which were used for the inside-out technique had a cost of $35 per patient according to 2012 values.

At the final follow-up visit, the mean Tegner score had improved from a preoperative score of 2.1 to 5.8 (p<0.001). The mean Lysholm score improved from a preoperative score of 46.3 to 89.2 (p<0.001). (Table 2) The Tegner and Lysholm scores were lower in the ACL reconstructed patients than in the intact patients (p=0.014 and p=0.029 respectively). (Table 3)

**Table 2:** Comparison of the Lysholm knee scores and Tegner activity levels in the patients with meniscus repair preoperatively and at latest follow-up.

The measured scores	Preoperative	Postoperative	p
Tegner score ACL reconstructed ACL intact	2.1±1.1 1.8±0.9 2.9±1.0	5.8±1.9 4.8±1.7 6.9±1.7	<0.001
Lysholm score ACL reconstructed ACL intact	46.3±15.9 44.4±8.2 70.1±16.2	89.2±4.2 93.9±4.4 97.8±3.0	<0.001

**Table 3:** Comparison of the latest follow-up scores of the patients with meniscus repair associated with ACL reconstructed and intact patients.

Postoperative scores	ACL reconstructed	ACL intact	p
Tegner score	4.8±1.7	6.9±1.7	0.014
Lysholm score	93.9±4.4	97.8±3.0	0.029

## DISCUSSION

The outcome of combined meniscal repair technique in BHMT was successful in our study. All meniscus tears were healed except one patient who had ACL re-rupture. Inside-out and all-inside techniques were combined in this study to avoid the neurovascular complication risk so that the all-inside technique was used in the posterior part of meniscal tears and the inside out technique was used in the middle part of the meniscal tears because of technical simplicity and to reduce unexpected complications and to provide the cost-effectiveness.

Different success rates of meniscal repair have been reported from 66.1% to 100%^9^ ^,^ ^13^ ^,^ ^15^ ^-^ ^19^ but few reports have evaluated BHMT. Success rates of 83-89.6% have been reported after repair of BHMT.^12^ ^,^ ^13^ ^,^ ^19^ ^-^ ^21^ The high success rate of the current study can be considered to be due to some factors such as the vascularity of the meniscus, concomitant ACL reconstruction and fixation strength. The patients included in this study had red-red and red-white zone tears. Although the healing capacity of the white-white zone tears was low, O'Shea et al reported high healing success of white-white zone BHMT repair. He reported 5 failed in 43 repaired patients with BHMT.^12^ Furthermore, degenerative tears diminish the success rate and these patients were not included in the study. In combined repairs reported in literature, all have used the all-inside technique in posterior meniscal tears.^9^ ^,^ ^13^ ^,^ ^22^ However, with meniscal fixation devices, it has been reported that the success rates were low in posterior medial meniscal tears and this has been stated to be because of healing problems of the posteromedial meniscus.^13^ ^,^ ^23^

**Figure 3:**
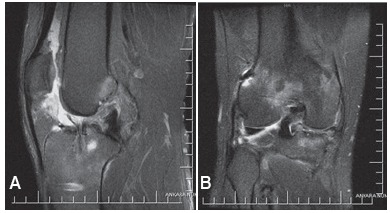
ACL re-rupture was seen 3 months after surgery on the sagittal view seen on MRI (A). The medial meniscus was intact and healed completely (B).

Many factors, such as a young age, acute tear, rim width less than 3mm, lateral meniscus tears and concomitant ACL reconstruction at the time of meniscal repair, influence the outcome of meniscal repair positively according to reports in literature.^2^ ^,^ ^3^ ^,^ ^19^ ^,^ ^24^ ^,^ ^25^Stone et al.^25^ stated that the time between injury and repair was the most important factor influencing healing. However in a recent published study revealed high success rate (83%) in repair of chronic BHMT according to Barrett's criteria at a mean follow-up of 48 months.^24^ No difference was determined in the Lysholm scores between red-red and red-white zone tears in the patients in our study (p=0.640). Morgan et al.^16^ reported a 92% failure rate for posterior medial meniscus tears and concluded that meniscal repair failure was strongly associated with an original location in the posterior horn of the medial meniscus, and that incomplete healing was also associated with posterior horn repair of the medial meniscus as well as Ahn et al.^22^ reported Potential reasons for a higher reoperation rate after repair of the medial meniscus include the fact that the medial side of the meniscus is anchored more tightly to the tibial plateau and that the medial side sees higher biomechanical loads^26^ However Ahn et al.^27^ reported only 3.6 % failed healing of medial meniscus posterior horn tears with ACL reconstructions Although there was complete recovery of all but one menisci in the current study, the Lysholm scores were compared to ascertain if there was any difference between medial and lateral meniscus repairs and no difference was found (p=0.205). The most important issue is that if residual laxity persists after ACL reconstruction, the medial meniscus may be exposed to greater stress because it is a secondary stabilizer to anterior tibial translation. This may put a repaired medial meniscus under more stress, potentially contributing to more failures as the menisectomized patient in our study. (Figure 3)

The all-inside technique is more expensive than the inside-out technique. The cost of the sutures used for BHMT was calculated. The cost of the FasT-Fix meniscal repair system which was used for the all-inside technique was found to be $335 per patient, whereas the Ti-cron sutures which were used for the inside-out technique had a cost of $35 per patient according to 2012 values. Furthermore, to the best of our knowledge, the operation time for the inside-out technique did not differ because of technical simplicity in the middle part of the tear. In addition, the all-inside technique of meniscus repair is technically more demanding as the torn part is closer to the anterior part of the meniscus.

Although ACL reconstruction leads to high healing rates, the Lysholm and Tegner scores of the meniscal repair patients with associated ACL reconstruction were lower than those of the patients with intact ACL (p=0.029 and p=0.014 respectively). This was thought to be due to the prolonged rehabilitation protocol and the possibility of higher levels of pain seen after surgery and related disuse atrophy in ACL reconstructed patients. In addition, rehabilitation protocol was more restrictive in associated ACL reconstructed tears and this could have been a contributing factor to the functional scores.

Although it has been reported that the successful results of meniscal repair patients deteriorate over time, the functional status of the patients are better than those of meniscectomized patients^1^ ^,^ ^25^The mean follow-up period in the current study was 31.3 months and long-term follow-up is needed to make a decision and this can be considered as a limitation of this study. Additionally, as this study was retrospective there was no control group, so no comparison could be made of the outcomes of the patients. Furthermore, the status of the meniscus could not be seen directly with second-look arthroscopy to give accurate results.

## CONCLUSION

Combined meniscal repair using the all-inside technique in the posterior part and the inside-out technique in the middle part of the meniscus is a successful and cost-effective treatment method in BHMT either with or without ACL reconstruction in one session. Although ACL reconstruction has a positive effect on meniscal healing according to the literature, the functional scores were lower than in patients with intact ACL with BHMT.
